# A survey of dental school's emergency departments in Ireland and the UK: provision of undergraduate teaching and emergency care

**DOI:** 10.1038/sj.bdj.2015.436

**Published:** 2015-06-12

**Authors:** S. Anderson, J. Nunn, L. F. A. Stassen, J. McLoughlin

**Affiliations:** 1 Lecturer in Emergency Dental Care, Dublin Dental University Hospital, Lincoln Place, Dublin 2, Ireland; 2 Professor of Special Care Dentistry, Dublin Dental University Hospital, Lincoln Place, Dublin 2, Ireland; 3 Professor of Oral and Maxillofacial Surgery, Dublin Dental University Hospital, Lincoln Place, Dublin 2, Ireland; 4 Director of Undergraduate Teaching and Learning, Dublin Dental University Hospital, Lincoln Place, Dublin 2, Ireland

## Abstract

**Aim** Emergency dental care is a vital service that new graduates should be prepared to offer. There are few published data relating to emergency dental care education. To assess this, and to gain a profile of accident and emergency departments (A&E) in dental schools, an online survey was sent to all of the dental schools in the Republic of Ireland and the UK.

**Setting** The survey addressed the school's A&E curriculum, teaching methods, undergraduate exposure and departmental details.

**Results** The majority of A&E departments operated during normal working hours with a minority offering an out-of-hours service. Teaching of A&E topics, and undergraduate experience, vary significantly between schools. A&E departments were diversely named and exhibited significant regional variation. Approximately half employed a triage system. It is unclear what represents an adequate level of undergraduate exposure, and more research is required in this area.

**Conclusions** Assessment of undergraduates following time in clinic is an important component of any A&E module. We consider a reflective portfolio to represent a suitable form of assessment, and would recommend their introduction. In addition, we recommend that dental hospitals consider a nurse-led triage system.

## Introduction

On graduation, dentists will see cases requiring emergency dental management. As and when dentists enter general practice after qualifying, they will have to deal with these cases independently, requiring both a sound theoretical knowledge and adequate practical experience.

In the UK, dental schools are regulated by the GDC. The GDC requires that dental schools ensure that students have demonstrated attainment across the full range of learning outcomes, which include several of particular relevance to dental A&E.^1^ The outcomes should encompass the development of skills and competence in the relevant field. The student should also be assessed to demonstrate fulfilment of these learning outcomes.^2^

In many cases, topics relevant to emergency dental care are covered in multiple, separate areas of the curriculum. Good practice should allow integration and consolidation of these topics to allow a holistic understanding of acute presentations. Undergraduates are most likely to gain experience of urgent dental care through their dental school's emergency clinic. This may be their only opportunity to gain experience of management of acute pain, facial swelling, facial trauma and other acute conditions before graduation.

There are no published data relating to A&E departments in dental schools in the Republic of Ireland or the UK. In 2007 Tiwana K *et al*. looked at emergency dental education in DDS courses in the United States.^3^ The authors conducted a paper survey with an 88% response rate, and found that the emergency dental care curricula were quite varied, and lacked experience of paediatric emergencies. They also found that the majority (65%) of US dental schools did not overtly evaluate the students' performance in the emergency setting.

The authors recommended that dental students have earlier exposure to emergency clinics to improve their understanding of acute dental needs. The literature does however contain papers on dental out-of-hours emergency care arrangements in the UK, illustrating regional diversity of out-of-hours arrangements.^4^,^5^,^6^,^7^,^8^,^9^,^10^

In the light of the paucity of data on the undergraduate experience of dental A&E, an online survey was designed, for distribution to the lead personnel in dental A&E in dental hospitals in the UK and Ireland. It focused on the emergency dental care curriculum, undergraduate exposure, provision of teaching, and on the emergency dental care clinic itself. The purpose was to gain a profile of each dental school's provision of emergency dental care teaching and their A&E clinic, including out-of-hours arrangements.

## Methods

A 30-question online survey was designed to gather information relevant to the undergraduate A&E curriculum, exposure of undergraduates to A&E clinics, teaching methodology in A&E topics, and operational details of A&E clinics. If a respondent did not answer a question, the survey design allowed respondents to progress to the next question. This resulted in different numbers of responses for several questions. We have reported the data from each question as a percentage of respondents to that question, not a percentage of the overall number of respondents.

Multiple answers were allowed on several questions, and several questions provided space for additional comments.

In March 2013 the survey was uploaded to surveymonkey.com, a questionnaire website. The deans of each of the two dental schools in the Republic of Ireland and the 16 dental schools in the UK were emailed requesting that they forward the survey web-link to the head clinician of their emergency department. Non-responders were then emailed again in April and May of 2013.

## Results

### Respondents

All 18 dental schools responded, giving a 100% response rate. However, one survey was incomplete, giving an effective response rate of 94% (17/18). Respondents included two deans, three professors, three consultants, one director of undergraduate dental studies, one associate specialist, four lecturers in the topic, two speciality dentists and two clinical teachers/lecturers. Respondents were asked to state their qualifications. One respondent chose not to provide information about qualifications; the remainder all had a primary dental qualification (BDS) as a minimum. Sixteen had additional qualifications including MFDS or equivalent, specialist qualifications such as FDS (Rest Dent), medical qualifications such as MBBS and FRCS, academic qualifications such as PhD, educational qualifications such as PGCE and MSc.

Survey questions, and their responses, were grouped into the following categories - curriculum, exposure, teaching and departmental.

### A&E curriculum

Of the 17 respondents to this part of the survey, 41% (7/17) did not require undergraduates to complete a dedicated dental A/E module.

The schools that did not have a dedicated A&E module (41%, 7/17) cited 'topics incorporated into other modules' as the main reason. No schools cited lack of financial resources, or lack of a perceived need for a dedicated module as a reason.

Of the seven schools that did not have an A&E module, 86% (6/7) reported they did not see a need for one. When asked why they saw no need, two respondents reported that relevant topics were taught elsewhere, two reported that A&E is incorporated into a general practice-type setting, one stated 'because students get experience of emergency care' and one stated that the issue has not yet been discussed.

**Figure 1:**
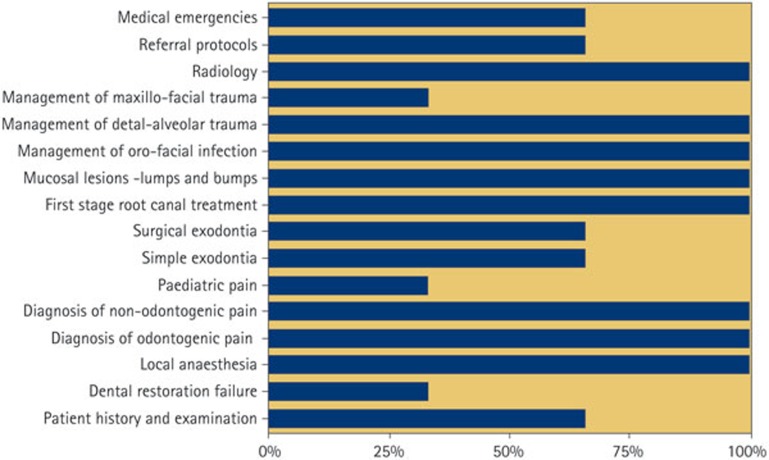
Please indicate which of the following topics are included in your school/hospital's dental A&E curriculum (ten respondents)

Of the ten schools that did have a dedicated module, 30% (3/10) had a written A&E curriculum available (Fig. 1).

**Figure 2:**
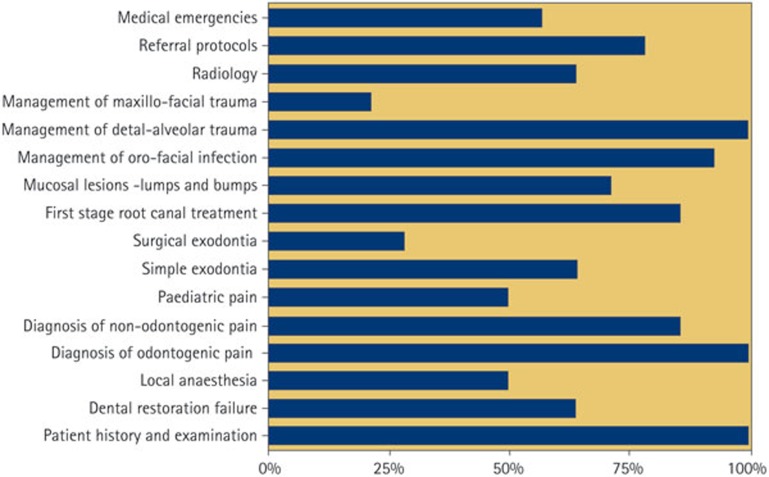
Which of the following topics you feel would be suitable for a dental A&E curriculum? (14 respondents)

Respondents who did not have a written A&E curriculum (82%, 14/17) were asked which topics they felt would be appropriate for inclusion in any proposed A&E curriculum (Fig. 2). Additional suggestions included prescribing protocols, behaviour management for the patient in pain and assessment, as opposed to management, of maxillofacial trauma.

### A&E teaching

Respondents were asked how topics related to A&E were taught - 16 responses were received and multiple responses were allowed. Topics of relevance to A&E are provided in 75% (12/16) of schools; of these, 69% (11/16) include seminars; 50% (8/16) include problem based learning; and 100% (16/16) report that teaching is delivered on clinics. Comments indicated that tutorials on clinics, multi-departmental symposia, outreach clinics and a blend of all suggestions were used.

Of the ten respondents that had a dedicated A&E module, 50% (5/10) reported that their A&E department's lead clinician was an oral surgeon; 30% (3/10) had a restorative dentist; and 20% (2/10) had an oral and maxillofacial surgeon (OMFS) as their lead clinician. Other lead clinicians included a dental and maxillofacial radiologist, an orthodontist and a special care dentist.

When asked which departments covered topics related to A&E, 15 responses were received. All reported that topics related to dental A&E were taught by more than one department. All but one, 93% (14/15), reported involvement of oral or maxillofacial surgery; 87% (13/15) involved restorative dentistry; 80% (12/15) included paediatric dentistry; 60% (9/15) included oral medicine. One respondent commented that dental A&E staff contributed to teaching in these topics.

### Exposure of undergraduates to dental A&E clinics

All undergraduate courses in the Republic of Ireland and the UK are five-years in duration. Our results indicate that all schools that have a dental A&E or equivalent (94% 16/17) require their undergraduates to attend these clinics. Only a minority require first and second years to attend dental A&E clinics, 12.5% (2/16) and 25% (4/16), respectively.

**Figure 3:**
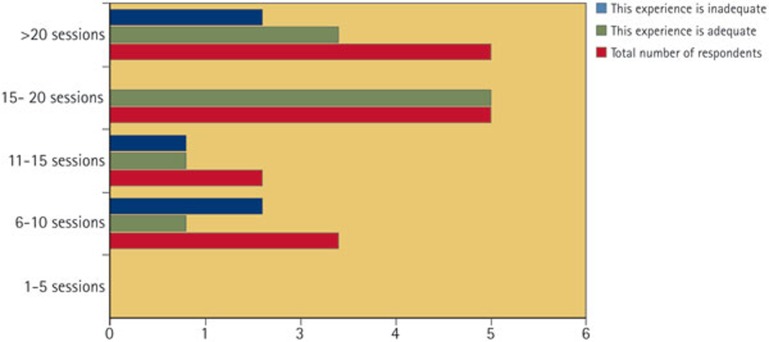
Level of undergraduate exposure (15 respondents)

When asked about exposure to dental A&E upon graduation, 15 responses were received. No respondents reported fewer than six sessions; 20% (3/15) of schools reported six to ten sessions; 13% (2/15) reported 11–15 sessions; 33% (5/15) reported 15–20 sessions; 33% (5/15) indicated more than 20 sessions. Respondents were then asked whether the level of experience was adequate or inadequate. These data were cross-tabulated with the number of sessions (Fig. 3). Overall, 33% (5/15) felt that the level of exposure upon graduation was inadequate.

Respondents were asked what grade of staff supervised undergraduates in A&E clinics. There were 15 respondents and multiple answers were allowed. Of the 15 respondents, 80% (12/15) were supervised by speciality grade dentists and 80% (12/15) by general dental practitioners. Oral surgeons supervised in 60% (9/15) of cases and house officers/senior house officers in 33% (5/15). Oral maxillofacial surgeons, endodontists and paediatric specialists were each reported as supervising in 6% (1/15) of places. Only one school had a dedicated dental A&E lecturer, who was responsible for supervising on clinics, and two reported that consultants in special care dentistry and maxillofacial radiology supervised.

The 16 schools that required undergraduates to attend dental A&E clinics were asked if they required students to complete a logbook or portfolio. Of the 15 respondents, 66% (10/15) required students to complete a logbook or portfolio.

Reportedly, students may also gain experience of dental A&E topics in the following areas: oral surgery clinics, outreach clinics, operating theatres, the paediatric clinics, general hospitals (particularly for OMFS emergencies), local anaesthetic clinics, restorative clinics and primary care clinics.

### Departmental

Only one of the 17 respondents reported that they did not have a dental A&E or equivalent. The 16 clinics were diversely named; three were named 'Accident and emergency', two were named 'Acute dental care', two were named 'Walk-ins', two were named 'Dental emergency clinic', two were named 'Primary care', and unique names include 'Oral diagnosis', 'Examination and emergency', 'Acute adult dental care' and 'Dental casualty'. One respondent did not provide a clinic name.

When asked if the A&E department employs a triage system, 15 responses were received. Of these 15, 47% (7/15) reported that they do not use a triage system at all; 40% (6/15) used a nurse-led triage system, all of which are pro forma based; 13% (2/15) used a dentist-led triage system, half of which are proforma based; no respondents reported using a secretary led system; one school reported using a central call-centre, which distributes patients to local practices as well as the dental hospital's emergency department.

Respondents were asked which clinicians provide the majority of the service in the department. There were 16 responses and multiple answers were allowed.

Respondents were asked which patients their department accepted. There were 16 response and multiple answers were allowed. All departments (16/16) accepted patients with symptoms of irreversible pulpitis; 94% (15/16) accepted patients with symptoms of reversible pulpitis; 88% (14/16) accepted patients with broken teeth without pain; 81% (13/16) accepted non-painful mucosal lesions; 69% (11/16) accepted de-bonded crowns/bridges.

**Figure 4:**
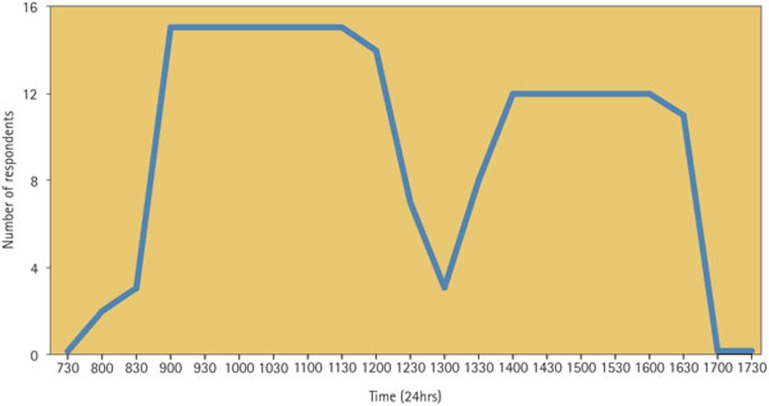
Please indicate the opening hours of your department during the week (Monday to Friday, not including out-of-hours) (16 respondents)

During the week, the majority of A&E departments (12/16) began seeing patients at 9am (Fig. 4). The majority reported closing for a lunch break beginning at 12.30pm and reopening at 1.30–2pm. Of the 16 respondents 25% (4/16) reported they were not open in the afternoon; one reported that they were only open for 'true emergencies (bleeding, trauma, sepsis with visible swelling)'; 19% (3/16) provided an out-of-hours (on-call) service at the weekend and on bank holidays. Of these three, two operated at night and one operated between 5–10pm; one of the three on-call services was for OMFS cases only.

## Discussion

The response rate was 100% (18/18), but unfortunately one survey was incomplete, giving an effective response rate of 94% (17/18). The responses to our survey indicated that the teaching and provision of emergency dentistry (dental A&E) varies significantly between different schools in the Republic of Ireland and the UK.

### Teaching undergraduates

In the UK the GDC is responsible for the quality assurance of dental education and training. There are two GDC documents that are of particular relevance to these areas.^1^,^2^ The GDC document '*The first five years*' has recently been superseded by the document '*Preparing for practice*'. With this change, the GDC has moved away from detailed prescription of specific topics, and towards an emphasis on learning outcomes and suitable assessment of these learning outcomes.

To work effectively in a dental A&E, a sound knowledge and clinical ability in virtually all fields of dentistry are required. The GDC's learning outcomes include some of particular relevance to A&E.^1^ The document '*Profile and competences for the European dentist*' also contains some competences which are particularly relevant.^11^ Any European dental A&E curriculum should meet the requirements of the regulating body in the country in which the course is delivered, and, ideally, lie within the framework of the '*Profile and competences for the European dentist*'.^11^

Students should be appropriately assessed against each of the learning outcomes.^2^ A wide variety of assessment methods may be used and these include continuous assessments, student portfolio, case presentations, written exercises, research exercises, peer feedback, as well as summative end of module/year/programme examinations. Student portfolios, training in and evidence of self-reflection, and evidence of mentoring and feedback may therefore represent part of the overall assessment.

### Curriculum

All respondents reported that topics related to A&E are taught by a variety of departments, and often incorporated into other modules. A module is defined as a 'learning unit, independent from the discipline or departmental structure'. Ideally, each module should have a manual at least describing the essential components.^12^

Given the importance of dental A&E as a topic, we suggest that a dedicated A&E module may help to consolidate the undergraduate's knowledge and increase their confidence in dealing with 'emergency' problems, which can be daunting for a new graduate.

Although ten schools required undergraduates to complete a dedicated dental A&E module, only three have a written curriculum. We would suggest that any school requiring completion of a defined A&E module should have a written curriculum complete with objectives, learning outcomes and a suitable assessment.

Interestingly, only one third of existing A&E modules included management of paediatric pain. Presumably the schools which did not include paediatric pain management incorporated this topic into another module such as paediatrics. Of the schools that did not have a dental A&E module, only half felt that paediatric pain management would be suitable for a future dental A&E curriculum. This may be because some schools have a separate clinic for management of paediatric emergencies.

Of the schools that did not have a dental A&E curriculum available, only 28% (4/14) felt that surgical exodontia was a suitable topic for a future dental A&E curriculum. However, the authors' experience of A&E would suggest that surgical exodontia is an essential competence in the department.

Only 33% (1/3) of current dental A&E modules included management of maxillofacial trauma. Of the schools that did not have a dental A&E curriculum available, only 21% (3/14) felt that maxillofacial trauma management would be suitable for a future A&E curriculum. It is possible that if the question had included 'assessment of maxillofacial trauma', a larger proportion of respondents would have thought it appropriate for inclusion in an A&E module. It could be argued that any clinician treating dental trauma should be competent in diagnosing fractures of the facial skeleton, as well as recognising signs of head injury, eye injury and cervical spine injury. Indeed, 'surgical extraction of an uncomplicated unerupted tooth and the uncomplicated removal of fractured or retained roots' and recognition of 'maxillofacial problems' are included as a competence in '*Profile and competences for the European dentist*'.^11^

### Teaching

All respondents reported that dental A&E topics are taught by a combination of methods, always including time on clinics. When asked which departments cover topics related to dental A&E, one respondent commented that dental A&E staff contributed. If this had been an option in the question, it is possible that more respondents would have included this group.

### Exposure

The GDC stipulates that students must only progress to treatment of patients when they have been assessed and deemed competent in the relevant area. They should then provide patient care under the supervision of an appropriately qualified and trained supervisor. The GDC stipulates that students should have adequate experience to develop skills and the level of competency to achieve their defined learning outcomes. It therefore seems essential that student's should have some exposure to dental A&E type clinics.

All schools that have an A&E department required students to attend. The one school that did not have a dental A&E department reported that students gained experience of these topics in general practice style clinics and maxillofacial surgery placements. Two thirds (10/15) of schools reported that students would have had at least fifteen sessions of A&E experience upon graduation. However, 33% (5/15) felt that the level of exposure gained was inadequate. Two of the five who reported over 20 sessions felt that even this level was inadequate - both of these respondents were from an oral surgery background. There is therefore great discrepancy in what respondents felt was an adequate level of experience upon graduation.

There was no requirement to complete a logbook or portfolio during time on dental A&E clinics for 33% (5/15) of respondents The literature is clear that a competency-based curriculum, as outlined in '*Profile and competences for the European dentist*', calls for competency-based assessment strategies. Portfolios are a suitable form of assessment strategy that takes learning experience to the next level through student reflection.^13^

### The A&E department

Defining the role of a dental A&E department can be challenging. A focus group of 20 GPs and GDPs agreed that true dental emergencies are quite rare.13 It was agreed that a 'dental emergency' should be seen within four hours of presentation, and consisted of:

- Haemorrhage
- Acute trauma to the teeth and jaw
- Swelling around the eye or swelling resulting in difficulty swallowing.

It was notable that the majority of dental A&E departments see patients with some of the 'lower priority' problems. In retrospect, the survey should have asked respondents to state whether or not they treated these problems with lesser urgency than true 'dental emergencies'. It may be that some schools offer appointments for these less urgent problems, a number of days after triage.

We were surprised that only 47% (7/15) of respondents employed a triage system. The medical profession has employed triage systems widely and with great success, finding that it increases doctor's efficiency and job satisfaction.^14^ Both telephone and nurse-led triage systems have been shown to be safe and effective.^15^,^16^ Indeed, one study has shown that a significant proportion of dental 'walk-in' patients would have been satisfied with verbal advice and a later scheduled appointment.^4^ We would therefore suggest that more dental A&E departments could benefit from an effective triage system.

Surprisingly few (19%, 3/16) schools offered a dental on-call service. The GDC guidelines for the provision of out-of-hours care are deliberately vague, to allow for interpretation tailored to local circumstances. It is generally agreed by the profession that treatment will be provided for registered patients within 24 hours and that a verbal response will be made within six hours.^17^ It is therefore difficult to comment on local arrangements, given the limited data available to us.

## Conclusion

The GDC documents '*Preparing for practice*' and '*Standards for education*', and the document '*Profile and competences for the graduating European dentist*', include several learning outcomes of particular relevance to dental A&E. The GDC are clear that students should have adequate exposure to ensure they have met these learning outcomes and should be assessed appropriately to ensure this.

Our survey indicated that great variation in the delivery of dental A&E teaching and in undergraduate exposure to A&E clinics exists. A more formal approach to dental A&E as a topic may be advisable.

Our survey also illustrated the considerable variation in the naming of dental A&E clinics, the 'emergency' conditions treated in the departments, the staff employed, the triaging of patients and the provision of an out-of-hours service.

In conclusion, consideration should be given, in any curriculum reform, to the implementation of a dedicated A&E module, which may help consolidate undergraduate's knowledge and increase their confidence and competence, based on a written curriculum with well-defined learning outcomes. This might usefully be supplemented with a reflective portfolio, mindful of the strain this imposes on staff resource. Further research into the optimum level of undergraduate exposure to dental A&E, and to the inclusion of a dental A&E module in an already crowded curriculum, is warranted.

## Notes

**Refereed Paper**
